# Trophic Enrichment Factors for Blood Serum in the European Badger (*Meles meles*)

**DOI:** 10.1371/journal.pone.0053071

**Published:** 2012-12-27

**Authors:** David J. Kelly, Andrew Robertson, Denise Murphy, Tara Fitzsimons, Eamon Costello, Eamonn Gormley, Leigh A. L. Corner, Nicola M. Marples

**Affiliations:** 1 Department of Zoology, School of Natural Sciences, Trinity College, Dublin, Ireland; 2 Centre for Ecology and Conservation, University of Exeter, Cornwall Campus, Tremough, Cornwall, United Kingdom; 3 School of Veterinary Medicine, University College Dublin (UCD), Dublin, Ireland; 4 Central Veterinary Research Laboratory, Department of Agriculture, Food and the Marine, Backweston, Celbridge, County Kildare, Ireland; University of Western Ontario, Canada

## Abstract

Ecologists undertaking stable isotopic analyses of animal diets require trophic enrichment factors (TEFs) for the specific animal tissues that they are studying. Such basic data are available for a small number of species, so values from trophically or phylogenetically similar species are often substituted for missing values. By feeding a controlled diet to captive European badgers (*Meles meles*) we determined TEFs for carbon and nitrogen in blood serum. TEFs for nitrogen and carbon in blood serum were +3.0±0.4‰ and +0.4±0.1‰ respectively. The TEFs for serum in badgers are notably different from those published for the red fox (*Vulpes vulpes*). There is currently no data for TEFs in the serum of other mustelid species. Our data show that species sharing similar niches (red fox) do not provide adequate proxy values for TEFs of badgers. Our findings emphasise the importance of having species-specific data when undertaking trophic studies using stable isotope analysis.

## Introduction

Stable isotope analysis [1] and the use of mixing models [2], [3] allow detailed investigations of the diet of wild animals. In combination they provide estimations of the contributions of different food sources to an animal's overall diet. As an animal metabolises the nutritional components of its diet, different isotopes of the same element are processed in different ways. In vertebrate species there is a tendency for heavier isotopes to be retained [4]. This process applies most notably to the metabolism of carbon and nitrogen, the essential building blocks of amino acids and proteins [5]. There are higher ratios of ^15^N to ^14^N and ^13^C to ^12^C in animal tissues compared to those of their food. This is referred to as trophic enrichment [2], [6] (synonymous with trophic fractionation, [7] enrichment, [8] fractionation [9] or discrimination [10]). The trophic enrichment factor (TEF) is a fundamental piece of information required by ecologists using stable isotope analysis [2], [3] to determine food sources and diversity, but TEFs have been determined for very few animals [11], [12], [13], [14]. For those species where TEFs have not been determined, researchers are forced to use values from trophically or phylogenetically related species [15], [16]. In the past few years, researchers have conducted stable isotope studies on mustelids without specific data for the species they were studying [17], [18]. Due to the lack of information in the literature, they have used animals as diverse as foxes, wolves, pigs and sea lions, as sources for TEFs. The use of proxy TEFs may be inappropriate, as TEFs may differ significantly between species of the same genus [19], between species in the same ecological niche [20], and a single species may show different TEFs depending on the specialisation of their diet [21], [22], [23].

To determine the TEF for a species, study animals must be maintained in a controlled environment, with a controlled diet, and for a prolonged period of time [24]. Ideally, animals should be fed on a single food source for a sufficient time to allow the isotope ratios in their tissues to equilibrate with their control diet. The time required to reach equilibrium varies between tissues, for example, the equilibration time for serum is faster than that for red blood cells (RBCs) [22]. After allowing sufficient time for equilibration between the tissue and the control diet, the TEF for each element can be estimated by a simple calculation: the isotopic value from the tissue minus the isotopic value from the food. However, it is not easy or convenient to maintain animals on a constant, controlled diet in a controlled environment [25]. While collecting tissues from animals across extended periods may assist in the interpretation of their diet [26], [27], such interpretations will only be qualitative and will lack sufficient detail for use in trophic mixing models. It is only when the TEF of the study animal is known, along with the isotopic values of the available food sources, that quantitative calculations may be undertaken [11], [22], [27].

The European badger (*Meles meles*) is a social mustelid found across Europe [28], [29]. In the UK and Ireland it is a reservoir for *Mycobacterium bovis*, the cause of tuberculosis (TB) [27], [30], [31], and hence the diet of badgers is of particular research interest in those countries [32], [33], [34], [35], [36]. Badgers are omnivorous [37], [38], [39], [40], opportunistic feeders [37], [41], showing evidence of seasonal specialisation [32].

The diet of wild badgers has been investigated using the analysis of stomach contents collected *post mortem* [41], [42] and the analysis of voided faeces [37], [43]. Recent work has demonstrated that the analysis of stomach contents of badgers gives a better indication of their diet than faecal analyses [44]. Stable isotope analysis of blood offers a complementary technique for the determination of badger diet. While gut contents and faecal analysis may provide information on the species being consumed, stable isotope analysis can provide information on the relative contributions of those species to the diet [45]. Blood sampling is a mildly invasive technique and offers a way of collecting time-series data on individuals. However, in order to generate the data from blood fractions, it is necessary to know the TEFs of those tissues.

Using captive badgers, fed on a controlled diet, we determined the TEFs for carbon and nitrogen in the serum (a blood fraction that turns over within a few days [46]). Lipid-extracted values were calculated for δ^13^C TEFs, as lipids have a lower δ^13^C values, relative to protein and carbohydrate [47], [48].

## Results and Discussion

### Lipid Extraction values for samples

Lipid extraction significantly changed the δ^13^C values of the dog biscuit and peanut samples, but not the blood serum (Table 1). We have provided data on the lipid-extraction of red blood cells (RBCs) (Table 1), although we were unable to generate reliable TEFs for that blood fraction. Where the lipid extraction process generated a significant change in the δ^13^C value, the differences were adopted as transformations and applied to all appropriate samples.

**Table 1:** Mean isotopic changes for δ^13^C after lipid extraction (including P values and sample sizes from comparison T-tests) of dog biscuits, peanuts and badger blood serum and badger red blood cells (RBCs).

	mean δ^13^C change (‰) (± sd)	effect of lipid extraction	sample size
dog biscuit	0.9 (±0.3)	P<0.01	5
peanut	3.1 (±0.2)	P<0.001	5
badger serum	n/a	NS	5
badger RBCs	−0.8 (±0.2)	P<0.001	5

Reported values are provided with only one decimal place, to reflect the accuracy and precision of the analytical machinery.

### Trophic enrichment values for serum

**Figure 1:**
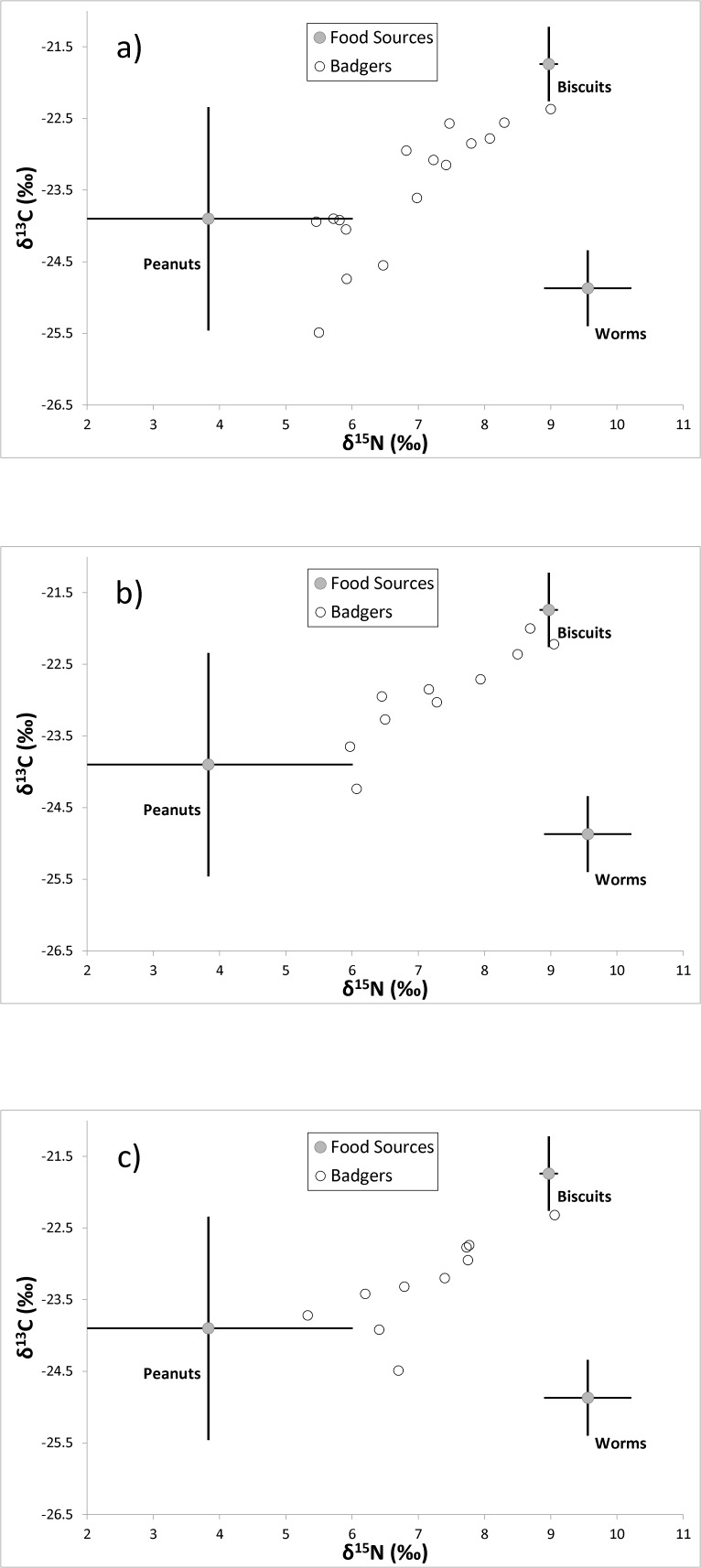
Stable isotopic values of serum from captive badgers fed a diet of biscuits and peanuts. These plots use trophic enrichment factors (TEFs) derived from three captive animals fed on a biscuit-only diet. The TEF for each dietary source is represented by a mean and standard deviation for both axes (δ^15^N and δ^13^C), plotted using SIAR [2]. Additional data for earthworms are provided for comparison. a. May 2008 (16 badgers). b. June 2008 (10 badgers). c. July 2008 (10 badgers).

The isotopic values of the biscuits and the badger serum samples from November 2008, after one month on a biscuit-only diet, are shown in Table 2. TEFs for δ^15^N and δ^13^C in serum were +3.0±0.4‰ and +0.4±0.1‰, respectively. These values were compared with serum data gathered from earlier in the year (between May and July 2008). The data for serum samples from May, June and July 2008 (Figures 1a, 1b and 1c, respectively) were plotted using Stable Isotope Analysis in R (SIAR) [2]. Figure 1 shows the isotopic values of the serum samples. No lipid-extraction adjustments were made to serum samples, as there was no demonstrable effect of lipid extraction (Table 1).

**Table 2:** Mean isotopic values for δ^15^N and δ^13^C (lipid extracted) obtained from serum of three captive badgers fed on a controlled diet for one month, and the calculated trophic enrichment factors (TEF) for each animal.

Sample	Badger	Isotopic Value (‰)		TEF (‰)	
		δ^13^C	δ^15^N	δ^13^C	δ^15^N
Serum	A	−23.6	9.6	0.5	2.5
	B	−23.6	10.3	0.5	3.3
	C	−23.7	10.1	0.4	3.0
mean(±sd)				0.4(±0.1)	3.0(±0.4)
Biscuit		−24.1(±0.3)	7.0(±0.1)		

Isotopic values (with corrections for lipid extraction) are the mean of three samples for each badger and nine samples of biscuit. Reported values are provided with only one decimal place, to reflect the accuracy and precision of the analytical machinery. TEFs for serum were calculated by subtracting the mean stable isotopic values of the diet (biscuits) from that of serum.

It is clear that the isotopic values of serum taken in May (Figure 1a), June (Figure 1b) and July 2008 (Figure 1c) are located roughly on a line joining the mean isotopic values of the biscuits and peanuts. These data support the calculated TEFs and suggest that the badgers were solely reliant on the provisioned food and were not supplementing their diet with other food available from the enclosures. Six earthworms sampled from the badger enclosures showed distinct isotopic values (mean δ^15^N = 6.6±0.7‰, mean δ^13^C = −25.3±0.5‰, n = 6), as did those from beetles (1 adult and 1 larva - data combined) sampled from the enclosures (mean δ^15^N = 7.7±0.2‰, mean δ^13^C = −26.3±0.7‰, n = 2). Invertebrate samples were corrected for lipids using a general invertebrate value [49]. It is clear that the captive animals were not ingesting large quantities of earthworms, beetles or beetle larvae, as their serum values would have been shifted away from the line between the peanut and biscuit values (Figure 1). This is an interesting finding, as earthworms have been considered to be an important part of the diet of badgers in the UK [39], [50] and Ireland [51]. However, we cannot exclude the possibility of a low level of ingestion of invertebrates.

### The potential of blood fraction TEFs for future work

In mammals, the half-life of RBCs is related to body size [52]. The body weight of an adult badger (6–17 kg [53]) is similar to that of a medium-sized dog. As the life span of RBCs in dogs is 90–135 days [54], we believe that the life span of RBCs in badgers would fall within a similar range.

The isotopic value obtained from serum is representative of the diet of an animal during the preceding week, based on serum protein turnover rates [22], whereas the isotopic value from the RBCs represents the diet during a period of three to four months [52], [54]. As badgers have a seasonally variable diet [32], [55], it seems unlikely that they would remain on a fixed diet for many months at a time. So, while the isotopic values of RBCs could provide information on longer-term (e.g. inter-annual) trophic shifts, studies using serum would probably be necessary to identify dietary changes within any given calendar year.

### Comparison of trophic enrichment values with other species

While there are data for TEFs of bone collagen [56], muscle [57], vibrissae [58] and RBCs [59], there is no data available on TEFs of blood serum for any mustelid species.

The red fox has a similar body weight range (3–14 kg [60]) to the European badger (6–17 kg [53]) and shares a similar trophic niche, being an omnivorous and opportunistic feeder [32], [37], [41], [55]. TEFs for serum in the red fox are +4.2‰ for δ^15^N [24] (higher than our value for badgers of +3.0‰) and +0.6‰ for δ^13^C [24] (slightly higher than our value for badgers of +0.4‰). The disparities in these data suggest fundamental differences in the metabolism of the two species, as both datasets used lipid-extracted samples derived from captive animals fed on a controlled diet of dried food.

We identified only one major difference between the red fox and badger protocols, being the sex ratio composition of the test groups. The red fox study (Roth & Hobson 2000) used groups with nine males and one female, whereas our study group comprised three females. While it is possible that there may differences in the TEFs of male and female badgers, we have found no indication that this has been recorded in other mammal species. Indeed, TEFs have been assumed to be the same for males and females when dietary differences have been identified between the two sexes [61], [62]. The red fox study group [24] and badger group 1 (this study) were both comprised of subadult animals.

### TEF variability with diet

It is probable that free-ranging European badgers will show clear variation in δ^15^N values when their diet has significant contributions from sources as disparate as carrion and fruit [32], [37], [40]. Our values for badger TEFs were derived from dried food with a mixture of meat, poultry and fish meal. It is likely that TEFs derived from frugivorous badgers will differ [11]. However, we were able to detect, using the TEFs we calculated for badgers fed on a controlled (biscuit) diet, the high proportion of peanuts in the diet of other captive badgers (Figure 1). This supports the validity of our experimentally-derived TEFs for serum and demonstrates they may be safely applied to badgers on an omnivorous diet.

### TEF variability with age

Roth & Hobson [24] showed that the TEFs of δ^15^N for subadult red foxes are greater than those for adult red foxes for liver, muscle and fur. Unfortunately, that study has no comparable data for blood fractions. In our study of blood serum, we have no directly comparable data for subadult and adult badgers, as all badgers in our study were subadults. If badgers exhibit the same trend as red foxes, our data would be shifted right on the X (δ^15^N) axes of the isotope plots (Figure 1), a result of subtracting a lower TEF from the δ^15^N values of the adults. As this would take the data further from an imaginary line joining the mean isotopic values for the provisioned food sources, and the expected range of dietary mixtures, we do not believe that the TEF of δ^15^N in blood serum for subadult badgers is greater than that of adult badgers.

## Conclusions

Knowledge of the TEFs for the serum of badgers should allow stable isotopic investigation of their trophic biology to be conducted in much greater detail. Our data, and its comparison with other species, demonstrates the risks that may be encountered when ecological similarities are employed to “guesstimate” the TEFs for a species where specific data are unavailable. We found significant discrepancies between the TEFs of badgers and species occupying a similar ecological niche (red fox). There appears to be no satisfactory substitute for empirical studies to determine TEFs for the tissues of a particular species [11]. While it may prove perplexing for trophic ecologists, a clear understanding of the differences in TEFs between animals of similar phylogeny, size and diet will serve to improve our understanding of their specific biologies.

## Methods

### Ethical statement

The studies on badgers were approved by the Animal Research Ethics Committee at University College Dublin and carried out under licence by the Department of Health and Children, Ireland (B100/3187).

### Animals

The badgers were held in outdoor pens, each ∼200 m^2^ with earthen floors covered in grass and shrubs. The group size within the pens varied from 1 to 4 animals. While 17 badgers were available for study between April and October 2008, only three individuals (all females, born in captivity in 2008), constituting one group, (group 1) were available through October and November 2008. By October 2008, all three of these animals had reached weights comparable with adult females in the wild (data from Department of Agriculture, Food and the Marine).

### Dietary variation

Between May and November 2008, the badgers were provided with a mixture of dog biscuits (Connolly's Red Mills, Goresbridge, Ireland) and shelled peanuts (Murtagh & Sons, Ashbourne, Ireland). Samples of peanuts and biscuits were taken for analysis from each new batch as they were used. Access to water was *ad libidum*. For the month of October 2008, the three badgers of group 1 were fed only dog biscuits. Biscuits were selected as the single-source diet as they were preferred to peanuts and showed less variation in their isotopic values (Figure 1).

### Blood Sampling

Blood serum was collected routinely (every four to five weeks) from each animal between May and November 2008. This sampling protocol allowed for a minimum of four half-lives of serum between samples [46]. Serum samples collected in November (after five weeks on a single-source diet) were used to calculate the TEF for blood serum. This value was compared to values obtained from serum collected earlier in the year, when animals had access to two food sources, dog biscuits and peanuts. Any changes to the diet of the badgers took place after the collection of blood samples.

### Invertebrate Sampling

A range of invertebrates, earthworms (Lumbricidae) (n = 6), beetle larvae (n = 1) and adult (n = 1) beetles (Carabidae), were sampled from the badger enclosures in October and November 2008. These invertebrates represented potential alternative food available to the badgers.

### Sample preparation

Blood samples were collected in serum separator tubes (SST, BD Vacutainer®) and centrifuged at 1400 g for 10 mins to separate the serum from the RBCs and stored at −20°C. The blood fractions were dried at 60°C for 24 h then ground into a fine powder with a mortar and pestle. Invertebrate, peanut and biscuit samples were similarly dried and ground. Analysis of serum was performed in triplicate, while ten samples of the peanuts and biscuits were used to derive mean isotopic values. All invertebrate samples were analysed only once. Analysis was conducted in a Thermo Delta^plus^ Continuous Flow Isotope Ratio Mass Spectrometer (CFIRMS) with a CE Instruments 1112 Flash Elemental Analyser. Nitrogen and carbon isotope ratios are reported relative to primary standards, atmospheric air (VAIR) and Peedee Belemnite (VPDB), respectively, using a working standard, l-Alanine (δ^15^N = −7.767‰, δ^13^C_organic_ = −25.119‰). Our isotopic values are reported to one decimal place, to reflect the precision and repeatability of the analytical technique.

### Lipid Extraction

As lipid is depleted in δ^13^C relative to protein and carbohydrate [47], [48], lipid was extracted from subsamples of the blood fractions and food sources. Using five paired replicates, lipid-extracted samples of dog biscuit, peanut and serum were compared to untreated (no lipid extraction) samples. By comparing the δ^13^C values of the untreated and lipid-extracted samples, a series of correction factors were identified.

Lipids were extracted from dried, homogenised samples using a Soxholet apparatus with a refluxing 2∶1 chloroform∶methanol solution [63]. The lipid-extracted samples were dried in an oven at 60°C until mass remained constant and ground to a fine powder. Carbon (δ^13^C) and nitrogen (δ^15^N) isotope values were determined using a Fissons 1108 elemental analyser (EA) linked to a continuous-flow isotope-ratio mass spectrometer (Isoprime – GV instruments). These analyses were conducted at the Food and Environment Research Agency mass spectrometry facility in York, UK.
